# Subcutaneous Sarcoidosis

**DOI:** 10.7759/cureus.49501

**Published:** 2023-11-27

**Authors:** Dedeepya Gullapalli, Avinash Vangara, Sandhya Kolagatla, Subramanya Shyam Ganti, Jayaramakrishna Depa

**Affiliations:** 1 Internal Medicine, Appalachian Regional Healthcare, Harlan, USA

**Keywords:** steroids, disease-modifying antirheumatic drugs (dmards), hilar adenopathy, cutaneous granulomatous disease, cutaneous sarcoidosis

## Abstract

This case report focuses on a 40-year-old female with multiple subcutaneous skin nodules presenting to the clinic for worsening skin lesions associated with erythema and mild tenderness. A biopsy of the skin lesions showed non-necrotizing granulomas with multinucleated giant cells. The patient was being worked up for non-necrotizing granulomatous skin lesions and was diagnosed with subcutaneous sarcoidosis. Sarcoidosis diagnosis is based on clinical presentation, histopathological changes, and ruling out other granulomatous causes. Our patient is being treated with systemic steroids, hydroxychloroquine, methotrexate, and adalimumab. The patient is nine months into the treatment. A clinically significant reduction in the nodule size was noted. Other systemic involvement of sarcoid was ruled out. This subcutaneous skin involvement is a rare finding called the Darier-Roussy sarcoid. Usually self-resolving but extensive, deformative lesions need to be treated.

## Introduction

Sarcoidosis is a systemic disorder that forms non-caseating granulomas in multiple body organs. Subcutaneous sarcoidosis or Darier-Roussy syndrome is a rare cutaneous manifestation of sarcoidosis [1]. Cutaneous manifestations vary from papules, plaques, lupus pernio, and erythema nodosum to subcutaneous nodules [2]. The typical subcutaneous nodules are mobile, firm, and painless. These nodules are restricted to the trunk and extremities [3]. Cesar Boeck described sarcoidosis of the lymph nodes and skin in 1899. Darier and Roussy described subcutaneous nodules on the trunk and limbs of six women in 1906 [4]. It is thought that environment and genetics play a role in the etiology of sarcoidosis. A fluorodeoxyglucose (FDG) positron emission tomography (PET) scan gives us a better insight into systemic involvement with subcutaneous nodules and helps in differentiating sarcoidosis from malignancies [2]. The peak incidence of cutaneous sarcoidosis is in the fourth decade. A positive family history has a 3.7-fold increase in risk. It is important to biopsy the lesion as it will aid in diagnosis [5].

## Case presentation

**Figure 1:**
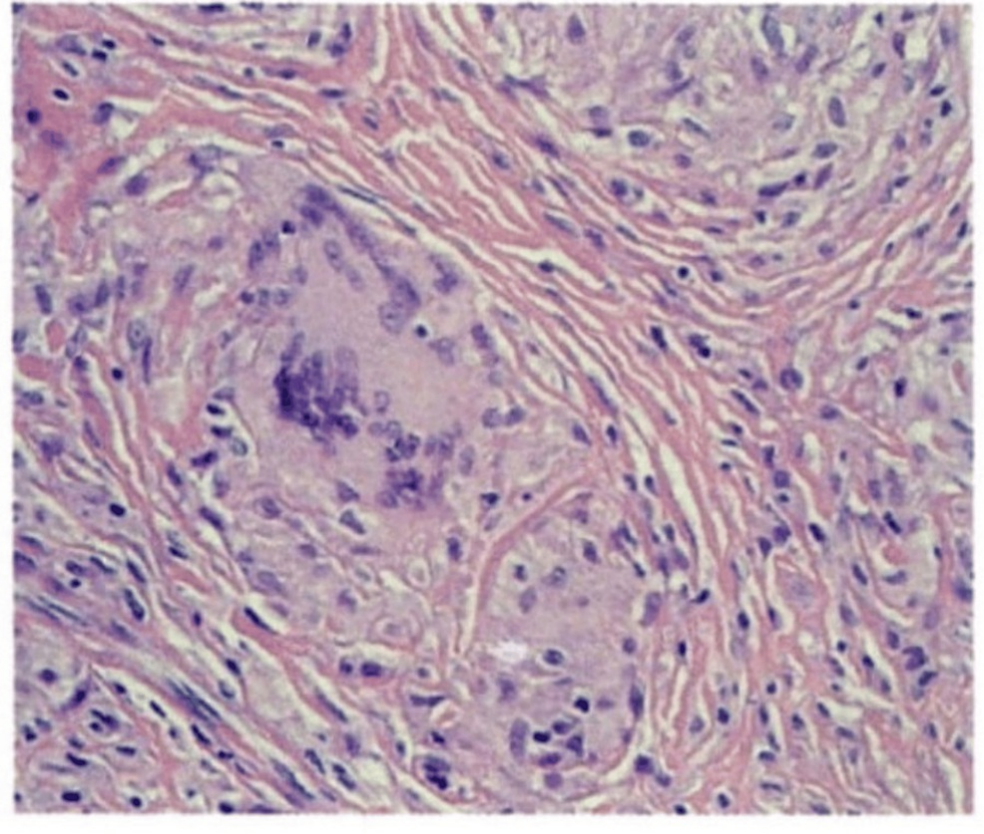
Multinucleated giant cells and non-caseating granuloma seen

A female in her 40s presented to the clinic with multiple soft tissue swellings in her bilateral upper extremities for four months. These swellings encompassed the forearms and bilateral elbows, involving the extensor aspects of her fingers and thumb. The condition has been progressively worsening, showing minimal erythema and mild tenderness. She does not recall having any trauma or infection. Her routine hematologic and biochemical laboratory tests were normal. Biopsy of the subcutaneous tissue showed numerous packed subcutaneous non-necrotizing granulomas with well-formed multiple multinucleated giant cells (Figure 1) and was negative for malignancy, acid-fast bacilli, and fungal organisms.

**Figure 2:**
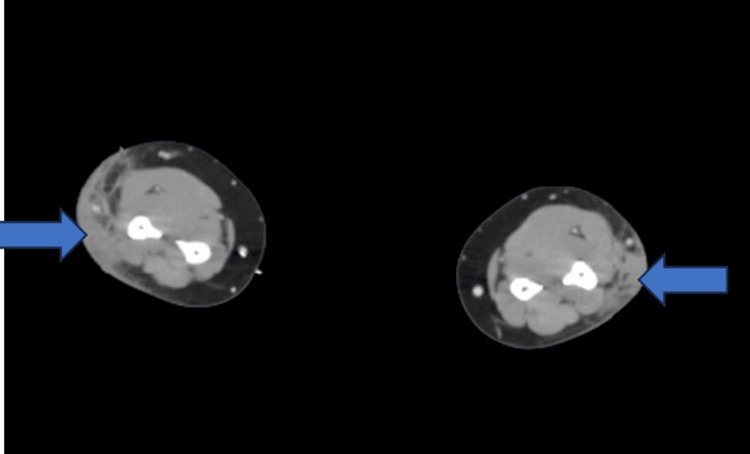
CT forearm showing subcutaneous nodules in the bilateral forearms with infiltrative changes (arrows showing the infiltrative changes)

The tuberculin skin test was negative. The anti-nuclear antibody screen, cyclic citrullinated peptide test, and rheumatoid factor were negative. Angiotensin-converting enzyme (ACE) levels resulted in 53 U/L (normal: 14-82 U/L). CRP of 4.3 mg/dl and ESR of 31 mm/hr were both elevated. 1,25-dihydroxy vitamin D was elevated at 105 pg/ml, but serum calcium was 9.6 mg/dl (normal: 8.5-10.1 mg/dl). A CT scan of the bilateral forearm showed infiltrative changes, and no abnormal enhancement within the soft tissues was seen (Figure 2).

**Figure 3:**
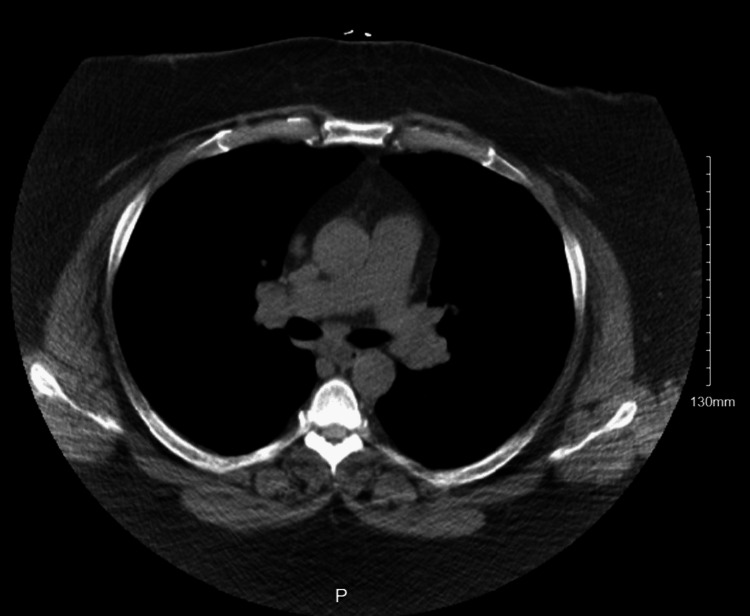
CT chest showing bilateral hilar adenopathy

CT chest showed hilar adenopathy with no interstitial changes (Figure 3).

Considering the differentials for non-necrotizing granulomas with multinucleated giant cell-like infections, these were ruled out by conducting gram staining and testing for fungal infections. An autoimmune workup was done to rule out immune-mediated granulomas. Sarcoid being the diagnosis of exclusion, other lab work like 1,25-dihydroxy vitamin D and ACE levels were done, where an elevated 1,25-dihydroxy vitamin D was noted. Other imaging was done to rule out multiorgan involvement. CT chest showed several calcified and non-calcified granulomas/nodules measuring <6 mm, and no interstitial changes in the lung were noted. Our patient was started on 10 mg of prednisone for eight to nine months by her primary care doctor for chronic bronchitis, and only a minimal reduction of the swelling was noted. After diagnosis, she was started on hydroxychloroquine 200 mg twice a day. Two months after starting the hydroxychloroquine, she was initiated on methotrexate 10 mg weekly along with folic acid, and a follow-up with a rheumatologist was made. Three months later, she was started on adalimumab (Humira CF) 4 0 mg subcutaneously every other week as her skin lesions were not subsiding. The patient had a positive response, and a reduction in the size of nodules was noted with adalimumab.

## Discussion

Clinical manifestations of sarcoidosis range from asymptomatic to progressive and relapsing [5]. Subcutaneous sarcoidosis is a rare manifestation of skin involvement affecting between 1.4% and 6% of patients with systemic sarcoidosis [2]. Subcutaneous sarcoidosis incidence is predominant in females, especially in the fourth decade, and presents with asymptomatic to slightly tender lesions. Another risk factor is family history, where incidence increases if it is seen in a first-degree relative, increasing the risk to 3.7-fold [5]. It can be associated with uveitis, arthritis, mucositis, dactylitis, hepatosplenomegaly, and neurological and renal involvement [6]. Serum ACE levels and 1,25-dihydroxy vitamin D are usually elevated. In an X-ray or CT scan of the chest, it is common to see bilateral hilar lymphadenopathy with or without mediastinal adenopathy [7]. Our patient had subcutaneous nodules; the CT chest showed lymphadenopathy but no lung involvement.

Sarcoidosis diagnosis comes with three key criteria: consistent presentation, non-necrotizing granuloma in one or more locations in histopathology, and elimination of other granulomatous diseases [5]. Differential diagnosis is discussed in Table 1. Cutaneous sarcoid presents as papules, plaques, erythema nodosum, lupus pernio, scar-associated sarcoidosis, and occasionally as subcutaneous nodules, i.e., subcutaneous sarcoidosis [2,8]. As sarcoidosis is a diagnosis of exclusion, a biopsy of the lesion and a punch biopsy for skin involvement are needed. It reveals noncaseating granulomas with a central collection of epithelial macrophages and multinucleated giant cells surrounded by sparse lymphocytes. This presentation of sparse lymphocytes is described as a naked granuloma. Our patient had an early initial presentation with deep nodules, and the histology showed numerous packed subcutaneous non-necrotizing granulomas. Multiple multinucleated giant cells were noted. A gram stain on the biopsy is conducted to rule out infectious etiology, while imaging is performed to rule out the involvement of other organs. Ultrasound examination can be considered a useful modality in monitoring the treatment [9]. Initially, hypoechogenic areas in subcutaneous tissue in later stages become hyperechogenic because of the repair process and fibrosis. Different echogenicity is seen in different stages of subcutaneous sarcoid. A differential of malignancy and other vascular malformations should be considered during initial ultrasonography [9]. The FDG PET scan helps in identifying other organ involvement and the response to treatment in complicated cases. It also helps in differentiating between subcutaneous sarcoidosis and malignancy [2]. According to the guidelines for disfiguring subcutaneous sarcoid, initially, intralesional steroid, followed by oral corticosteroid, is the first approach [2]. Our patient has multiple skin lesions for which intralesional steroid administration is recommended. The patient was on 10 mg of prednisone for eight to nine months for her chronic bronchitis [10]. The mainstay of treatment is systemic steroids (20-40 mg/day) when there is systemic involvement, and responses were noted within four to eight weeks after initiation of therapy. However, there is no specific treatment length, but the resolution is always the endpoint. In our patient, we did not do high-dose steroids as she is morbidly obese and worried about weight gain. Considering the complications of chronic steroid use, the most common alternate treatment used is hydroxychloroquine. Hydroxychloroquine alters the antigen presentation to CD4 T cells [2]. In vitro studies demonstrated inhibition of macrophage and other inflammatory cytokine production, particularly TNF-a, IL-1, IL-6, and IFN-g [11]. Methotrexate, clofazimine, intralesional glucocorticoids, thalidomide, dapsone, allopurinol, and minocycline are also used [12]. More prospective randomized control trials should be done on the hydroxychloroquine treatment observation. Early diagnosis is important for the treatment and prevention of progression to systemic disease.

**Table 1:** Differential diagnosis for non-necrotizing granulomatous disease

Skin manifestation	Differentiating findings
Foreign body	Multinucleated giant cells, polarization
Silicosis	Fibrotic nodules with an onion-skinned arrangement of collagen fibers
Infections	Positive stains for microorganisms
Sarcoidosis	Noncaseating granulomas

## Conclusions

Sarcoidosis, being a diagnosis of exclusion, is diagnosed by the clinical presentation and non-necrotizing granulomas in histology. One should always check the lung and other systemic involvement when thinking about sarcoidosis. No fixed duration of treatment for subcutaneous sarcoidosis has been hypothesized.

## References

[1] Vainsencher D, Winkelmann RK (1984). Subcutaneous sarcoidosis. Arch Dermatol.

[2] Youn P, Francis RJ, Preston H, Lake F (2022). Subcutaneous sarcoidosis (Darier-Roussy sarcoidosis) with extensive disease on positron emission tomography: a case report and review of the literature. Respirol Case Rep.

[3] Heller M, Soldano AC (2008). Sarcoidosis with subcutaneous lesions. Dermatol Online J.

[4] Young RC Jr, Rachal RE, Cowan CL Jr (1984). Sarcoidosis--the beginning: historical highlights of personalities and their accomplishments during the early years. J Natl Med Assoc.

[5] Sharma S, Adhikari A, Yadav SK, Mainali G, Rajkarnikar R (2022). Darier Roussy subcutaneous sarcoidosis from Nepal: a case report. Ann Med Surg (Lond).

[6] Ohashi T, Yamamoto T (2015). Subcutaneous sarcoidosis with underlying intramuscular granuloma. Indian J Dermatol.

[7] Celik G, Ciledag A, Akin P (2018). Subcutaneous sarcoidosis with plantar involvement. Ann Dermatol.

[8] Jadotte YT, Abdel Hay R, Salphale P, Mocellin S, Kumar S, Niazi A, Pilati P (2018). Interventions for cutaneous sarcoidosis. Cochrane Database Syst Rev.

[9] Dybiec E, Pietrzak A, Bartosińska J, Kieszko R, Kanitakis J (2015). Ultrasound findings in cutaneous sarcoidosis. Postepy Dermatol Alergol.

[10] Yamaguchi S, Shinoda K, Taki H, Hounoki H, Okumura M, Tobe K (2013). Systemic sarcoidosis with subcutaneous lesions in an 85-year-old female. J Am Geriatr Soc.

[11] Marchetti M, Baker MG, Noland MM (2014). Treatment of subcutaneous sarcoidosis with hydroxychloroquine: report of 2 cases. Dermatol Online J.

[12] Shigemitsu H, Yarbrough CA, Prakash S, Sharma OP (2018). A 65-year-old woman with subcutaneous nodule and hilar adenopathy. Chest.

